# The Transcriptional Role of Vitamin A and the Retinoid Axis in Brown Fat Function

**DOI:** 10.3389/fendo.2020.00608

**Published:** 2020-09-18

**Authors:** Carsten T. Herz, Florian W. Kiefer

**Affiliations:** Clinical Division of Endocrinology and Metabolism, Department of Medicine III, Medical University of Vienna, Vienna, Austria

**Keywords:** vitamin A, retinoid, obesity, brown fat, adipose tissue browning, thermogenesis

## Abstract

In recent years, brown adipose tissue (BAT) has gained significance as a metabolic organ dissipating energy through heat production. Promotion of a thermogenic program in fat holds great promise as potential therapeutic tool to counteract weight gain and related sequelae. Current research efforts are aimed at identifying novel pathways regulating brown fat function and the transformation of white adipocytes into BAT-like cells, a process called “browning.” Besides numerous genetic factors some circulating molecules can act as mediators of adipose tissue thermogenesis. Vitamin A metabolites, the retinoids, are potent regulators of gene transcription through nuclear receptor signaling and are thus involved in a plethora of metabolic processes. Accumulating evidence links retinoid action to brown fat function and browning of WAT mainly via orchestrating a transcriptional BAT program in adipocytes including expression of key thermogenic genes such as uncoupling protein 1. Here we summarize the current understanding how retinoids play a role in adipose tissue thermogenesis through transcriptional control of thermogenic gene cassettes and potential non-genomic mechanisms.

## Brown Adipose Tissue and Browning of White Fat

Brown adipose tissue (BAT) is an adipose organ specialized in producing heat to maintain body temperature. Brown adipocytes, in contrast to white adipocytes, are rich in mitochondria and are characterized by a large number of small multilocular lipid droplets as compared to unilocular lipid droplets in white adipocytes (1). The mitochondria of brown adipocytes express uncoupling protein 1 (UCP1) in the inner mitochondrial membrane which, when activated, uncouples the proton motive forced generated by mitochondrial oxidative metabolism from ATP synthesis and thereby dissipates chemical energy as heat (1). Promotion of brown fat thermogenesis counteracts obesity and related complications in numerous animal models and has therefore evolved as a promising novel therapeutic concept in the fight against the human obesity epidemic (2). Classical BAT depots, as most comprehensively described in rodents, embody mainly interscapular, axillar, cervical, femoral, and perirenal depots (1). However, brown-like or so-called beige adipocytes can also be found in white adipose tissue (WAT) depots, predominantly in subcutaneous fat and to a lesser extent in visceral fat (3). Stimulation of BAT thermogenesis classically occurs through hypothalamic noradrenergic signaling via the β3-adrenergic pathway in response to cold (1). This results in activation of protein kinase A (PKA) which promotes intracellular lipolysis and acts through the p38 MAPK as well as the CREB pathway which increases the expression of genes essential for the maintenance of thermogenic function such as UCP1, DIO2, and PGC1α (4). The emergence of beige adipocytes in WAT, coined “browning,” can occur in response to various stimuli including a number of genetic factors, hormones and chronic cold exposure. Beige cells can possess characteristics of both, classic white and brown adipocytes. When activated, beige fat cells express significant amounts of UCP1 and contribute toward thermogenesis and energy expenditure (4). It remains a matter of debate whether these newly formed beige adipocytes stem from mature white adipocytes undergoing conversion to UCP1-expressing cells following thermogenic stimuli or if a pool of distinct precursor cells gives rise to beige adipocytes. Elegant lineage tracing studies in mice provided evidence for both theories (5, 6).

Whereas, the salutary metabolic effects of brown fat have been unequivocally demonstrated in rodents, the impact of BAT physiology on human energy metabolism and its relevance for metabolic disease is less well-understood. Currently, the gold standard for the detection and quantification of active BAT in humans is ^18^F-fluorodeoxyglucose positron emission tomography/computed tomography (^18^F-FDG PET/CT) (7). The most potent physiologic stimulus for BAT activation is cold exposure that results in significant uptake of ^18^F-FDG in thermogenically active BAT depots and correlates in many studies with increased energy expenditure (8–11). The inverse relationships between active BAT and the degree of obesity and age also supports a potential protective role of BAT in metabolic disorders in humans (8, 12–14). Cold-induced BAT activity is mainly found in deep cervical, supraclavicular, para-aortal, and some renal fat depots in human adults (15). However, BAT in humans is not an organ as easily delineated from WAT as *bona fide* murine BAT since it comprises a mixture of brown and white adipocytes (16). The emergence of unilocular adipocytes in brown fat depots, called BAT whitening, has been demonstrated in animal models of aging and obesity (17, 18) and can be experimentally induced by high ambient temperature and defective β-adrenergic signaling, resulting in brown adipocyte death and inflammation (19). In contrast, clinical studies have found that repeated cold exposure over the course of 2 to 6 weeks, successfully increased the amount of active BAT as evidenced by ^18^F-FDG-PET/CT imaging in lean and overweight individuals as well as patients with diabetes, respectively (9, 20–23). The observed changes in BAT mass were accompanied by reductions in body fatness and improvements in insulin sensitivity (9, 21, 23). These findings not only suggest that thermogenically active BAT can be recruited in humans but emphasize the potential for therapies targeted at BAT with the aim to re-establish relevant amounts in BAT-depleted states such as obesity or older age and thus reverse associated metabolic aberrations.

## Vitamin A and Retinoid Metabolism

**Figure 1:**
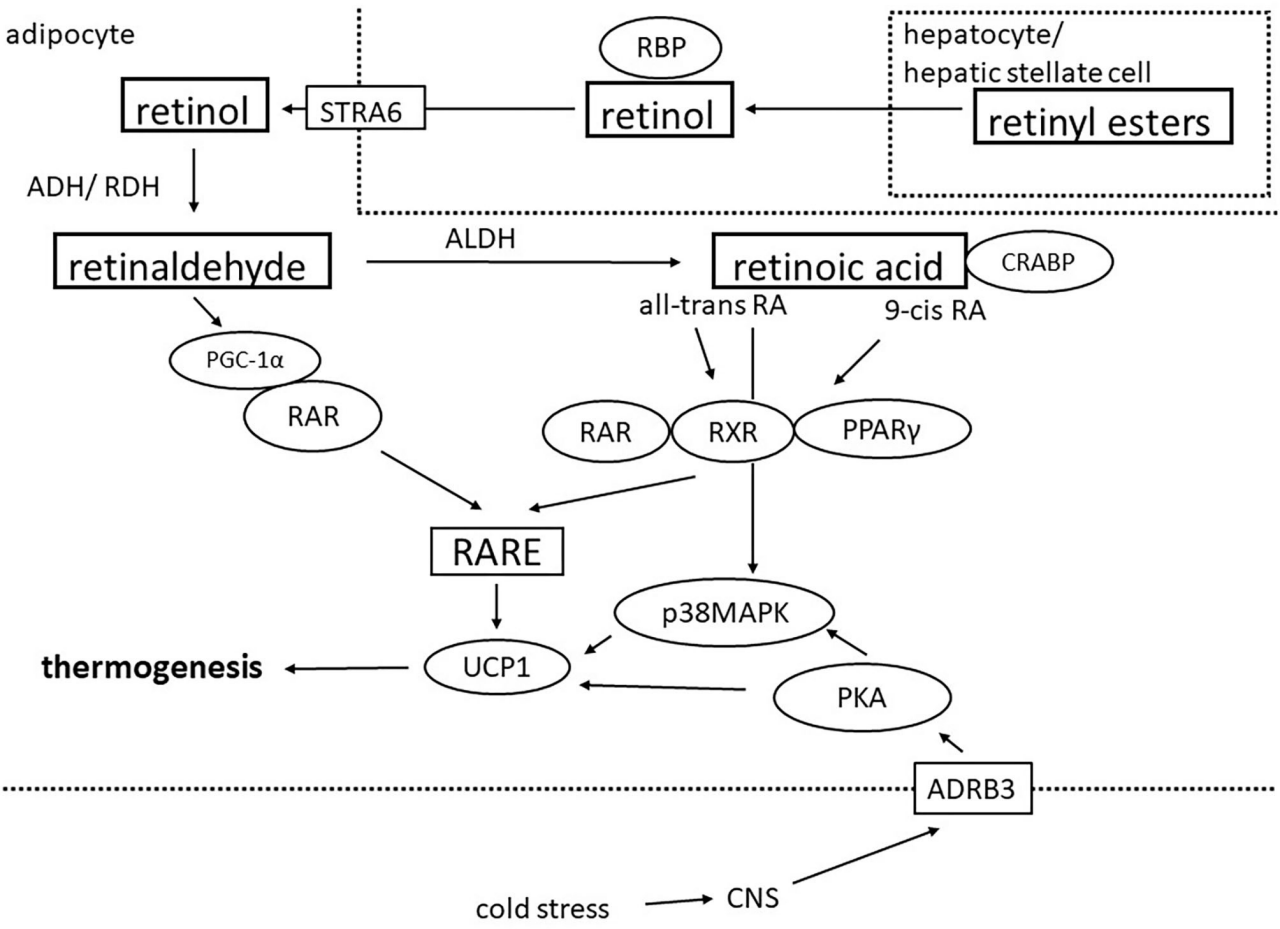
Schematic showing how retinoids regulate thermogenic gene expression in adipocytes. Retinoids are mainly stored as retinyl esters stored in hepatic stellate cells or hepatocytes and can be transported as retinol bound to retinol-binding protein (RBP) to peripheral tissues including adipose tissue. After uptake via the multi-transmembrane cell surface receptor STRA6, retinol is oxidized by alcohol- and retinol dehydrogenases (ADH/ RDH) to retinaldehyde which in the next step gets converted to retinoic acid (RA) by aldehyde dehydrogenases (ALDH). In the cytosol, retinoic acid is bound to cellular retinoic acid binding proteins (CRABP). Retinaldehyde and all-trans RA can activate the nuclear retinoic acid receptor (RAR) while 9-cis RA activates both RAR and retinoid X receptor (RXR). RXR also forms heterodimers with peroxisome proliferator-activated receptor gamma (PPARγ). RAR and RXR bind as homo- or heterodimers to genomic retinoic acid response elements (RARE) which can be found in the promoter region of the UCP1 gene and thereby regulate thermogenic gene expression. In addition, cold stress is the canonical activator of the thermogenic program in adipocytes via stimulation of the central nervous system (CNS). Efferent sympathetic neurons activate membrane-bound β3-adrenergic receptors (ADRB3) which leads to activation of the protein kinase A (PKA)-p38 mitogen-activated protein kinase (p38MAPK) pathway resulting in the transcription of UCP-1 and other thermogenic genes.

Besides their functions in cell differentiation, embryonic development, reproduction, retinal function and immunity, vitamin A and its metabolites, the retinoids, have been recognized as important regulators of energy metabolism (24). Vitamin A must be obtained from the diet by intake of either preformed retinol or provitamin A (carotenoids) which can be converted to retinol by beta-carotene monooxygenase. After absorption, the majority (~90%) is stored in the liver, while a smaller part (~10%) is stored in adipocytes (25, 26). In the liver, vitamin A is primarily stored in the form of retinyl esters in cytoplasmic lipid droplets of hepatic stellate cells (80–90%) and hepatocytes (10–20%) (25). Mobilization occurs via hydrolysis and binding to retinol binding protein (RBP) which transports retinol to the target tissues (25). In adipocytes, RBP-bound retinol is taken up by the multi-transmembrane cell surface receptor STRA6 (27). Intracellularly, retinol is then either re-esterified or converted to retinoic acid via two oxidative reactions: In the first step, retinol is reversibly oxidized to retinaldehyde (Rald) by alcohol– and retinol dehydrogenases (ADHs, RDHs) followed by irreversible oxidation to retinoic acid. The enzyme class of retinaldehyde dehydrogenases (RALDHs) has been identified to catalyze this rate-limiting step of retinoid metabolism. Intracellular retinoic acid availability and nuclear transport is facilitated by cellular retinoic acid-binding proteins and fatty acid binding protein 4 (28–30). Retinoic acid signals predominantly through the nuclear receptors retinoic acid receptors (RAR), retinoid X receptors (RXR) and peroxisome proliferator-activated receptors (PPAR) (24, 31) and is thus a potent regulator or gene transcription (Figure 1). While 9-cis retinoic acid has been found to be a potent ligand for RXR, its physiological relevance is under debate (32, 33). Quantification of 9-cis retinoic acid failed in most tissues of mice, rats and humans (34, 35). Despite a questionable physiological role, endogenous 9-cis retinoic acids or synthetic analogs might still be promising candidates for the activation of a thermogenic program in adipocytes through RXR, as discussed in the following section.

## Retinoids and Transcriptional Control of the Thermogenic Program

Accumulating evidence suggests that retinoids are involved in a number of metabolic processes including glucose and lipid metabolism, adipocyte differentiation and thermogenic programming of fat cells. Retinoid actions on metabolic pathways mainly depend on the regulation of gene expression through the nuclear receptor RAR and RXR which can also form RAR/RXR heterodimers. In addition, RXR works in concert with PPARγ, another key nuclear receptor controlling energy pathways and particularly adipocyte function (Figure 1). In 3T3-L1 cells, a murine model for white adipocytes, the effects of retinoic acid can vary dependent upon the stage of adipogenesis and expression of the transcription factors RAR, RXR, and PPARγ. Early in adipogenesis, retinoic acid inhibits whereas after 48 h of differentiation it promotes fat cell formation (36). The silencing mediator of retinoid and thyroid hormone receptors (SMRT) serves as a corepressor for nuclear receptors and regulates adipocyte differentiation, adipose tissue accumulation and insulin sensitivity. SMRT knockout mice have higher body weight on high-fat diet but increased insulin-mediated glucose disposal possibly due to a combination mechanisms involving an increased number of smaller subcutaneous adipocytes as well as decreased leptin expression, resulting in greater caloric intake (37). Some evidence suggests that retinoids can also act through non-genomic mechanisms such as protein retinoylation, a posttranslational modification shown to mediate cell differentiation, cell growth and possibly steroidogenesis (38). In recent years retinoids have been repeatedly linked to the transcriptional control of a brown fat program. Already in 1995, it was first reported that all-trans retinoic acid induced Ucp1 expression in murine brown adipocytes independent of differentiation status. Retinoic acid-response elements were found in the upstream region of the rat Ucp1 gene and RARα was identified as a mediator of the UCP1 responsiveness to retinoic acid (39–41) (Figure 1). However, studies showing that the RXR ligand 9-cis-retinioc acid also promoted Ucp1 expression in brown adipocytes to a similar extent as noradrenaline suggested that RXR may also be involved in inducing a BAT transcriptional program. Indeed, co-transfection of murine expression vectors for the different RAR and RXR subtypes indicated that RARα, RARβ, and RXRα are the major retinoid-receptor subtypes mediating the transcriptional response of Ucp1 to retinoids (42). PPARδ is another nuclear receptor regulated by all-trans retinoic acid with the potential to regulate BAT activity (43). In murine adipocyte cell lines, the effect of all-trans retinoic acid on thermogenic gene expression has however been shown to be independent of PPARδ (44). Retinoic acid may also alter the thermogenic capacity of brown adipocytes by non-genomic effects via induction of p38/MAPK phosphorylation (45). *In vivo*, the administration of both all-trans retinoic acid and 9-cis-retinioc acid markedly increased Ucp1 expression in brown fat depots in mice. 9-cis-retinoic acid even prevented BAT whitening through cold de-acclimation (46). In accordance, dietary supplementation of vitamin A in the form of retinyl acetate for 8-weeks significantly augmented Ucp1 expression in BAT of rats while decreasing the WAT marker leptin. Whole body adiposity was modestly reduced whereas feeding mice a retinol-deficient diet had the opposite effects (47, 48). Besides promoting thermogenic activity in *bona fide* brown adipocyte, retinoids also induce the emergence of brown-like thermogenic adipocytes in white adipose tissue depots: A four day subcutaneous treatment with all-trans retinoic acid in mice kept at thermoneutrality (30°C) which precludes sympathetic outflow to BAT and WAT due to cold stress resulted in higher expression levels of thermogenic genes as well as the appearance of multilocular adipocytes in the inguinal WAT depot (49). More recently, retinoic acid treatment in mice was shown to induce WAT browning by increasing adipose vascularity and promoting beige adipogenesis of platelet-derived growth factor receptor α positive adipose progenitors (50).

Besides retinoic acid, the precursor Rald has been identified as a signaling molecule in fat. Rald is essential in molecular vision processes, however a biological function outside the eye had long remained unknown. Work by Jorge Plutzky's group found that Rald is present in rodent WAT. *In vitro* stimulation with Rald inhibited white adipogenic differentiation in 3T3-L1 cells but markedly enhanced thermogenic gene expression in differentiated mesenchymal stem cells and primary human white adipocytes (51, 52). Rald treatment in murine adipocytes resulted in recruitment of the transcriptional co-activator Pgc1 to RAR present at the Ucp1 promoter. These transcriptional effects of Rald on thermogenesis were RAR-dependent. Mice deficient in Adh1a1, the enzyme converting Rald to retinoic acid, had elevated Rald levels in fat and were protected from diet-induced obesity due to increased energy dissipation. Mechanistically, Aldh1a1 deficiency promoted a thermogenic program in subcutaneous and even more so in visceral fat which rendered Aldh1a1^−/−^ mice cold resistant. This thermogenic phenotype was reversible when Aldh1a1 deficient mice were treated with an RAR antagonist. WAT-selective knockdown of Aldh1a1 by antisense oligonucleotides conferred a similar thermogenic program as in Aldh1a1^−/−^ mice, prevented diet-induced weight gain and improved glucose metabolism in mice suggesting that targeting Aldh1a1 in fat could be a potential therapeutic approach counteracting metabolic disease. Notably, Aldh1a1 is abundantly expressed in human visceral adipose tissue and increases with obesity (51, 52). In contrast, ablation of retinol dehydrogenase 1 (Rdh1) seems to have opposite effects. Rdh1 deficiency suppressed adiposity by promoting brown adipose adaptation to fasting and re-feeding. It has been shown that BAT activity is suppressed during fasting to preserve energy but it also contributes to diet induced-thermogenesis after food intake (10, 53, 54). Rdh1-null mice had lower body temperatures and a lower expression of Ucp1 in BAT. Mechanistically, Rdh1-deficiency resulted in decreased all-trans retinoic acid levels in BAT levels after refeeding which impaired lipolysis that is crucial for proper BAT function (55).

Whereas, all these data suggest that retinoids control thermogenic gene expression and BAT function, the retinoid pathways may also be regulated by cold exposure and adrenergic stimulation. The retinol transport protein RBP is induced by norepinephrine, cAMP and activators of PPARγ and PPARα in brown adipocytes. This effect requires the action of PPARγ-coactivator-1α and is absent in PPARα deficient adipocytes, suggesting that PPAR signaling is required of adrenergic induction of RBP in brown adipocytes (56).

All these reports show promise that retinoid pathways could serve as therapeutic targets to enhance energy expenditure and counteract obesity. Even though most reports stem from animal experiments, some *in vitro* studies in primary human adipocytes suggest that retinoids may also modulate thermogenic pathways in human fat (52). However, clinical studies on the association between retinoids and brown fat activity are lacking. Hence, validation of the previous preclinical findings in humans is warranted.

## Conclusion

Retinoids are vitamin A derivatives that are tightly regulated by a network of converting enzymes. Retinoic acid has been established as potent transcriptional regulator of thermogenic gene expression in adipose tissue, both, *in vitro* and *in vivo*. However, recent evidence suggest that retinoic acid is not the only biologically active vitamin A metabolite regulating thermogenic processes in adipocytes. Also the precursors, retinol and retinaldehyde may have independent biological functions in adipose thermogenesis. Targeting the retinoid pathway e.g., by interfering with retinoid converting enzymes that alter retinoid concentrations in selective tissues may offer novel therapeutic avenues to harness the energy dissipating qualities of BAT and beige fat for counteracting obesity and associated metabolic complications.

## Author Contributions

All authors listed have made a substantial, direct and intellectual contribution to the work, and approved it for publication.

## Conflict of Interest

The authors declare that the research was conducted in the absence of any commercial or financial relationships that could be construed as a potential conflict of interest.
